# Pre-evaluation assessment of serological-based COVID-19 point-of-care lateral flow assays in Kenya

**DOI:** 10.4102/ajlm.v10i1.1317

**Published:** 2021-09-17

**Authors:** James H. Kimotho, Abdiaziz A. Gosar, Ronald Inyangala, Paulyne Wairimu, Fred Siyoi, Damaris Matoke-Muhia, Cecilia Wanjala, Jeremiah Zablon, Moses Orina, Lucy Muita, Jacqueline Thiga, Lameck Nyabuti, Eunice Wainaina, Joseph Mwangi, Alice Mumbi, Samuel Omari, Ann Wanjiru, Samson M. Nzou, Missiani Ochwoto

**Affiliations:** 1 Innovation Technology Transfer Division, Kenya Medical Research Institute, Nairobi, Kenya; 2 Pharmacy and Poisons Board of Kenya, Nairobi, Kenya; 3 Centre of Biotechnology Research Development, Kenya Medical Research Institute, Nairobi, Kenya; 4 Kenyatta National Hospital, Nairobi, Kenya; 5 Centre for Virus Research, Kenya Medical Research Institute, Nairobi, Kenya

**Keywords:** COVID-19, point-of-care, IgM, IgG, sensitivity, specificity, SARS-CoV-2

## Abstract

**Background:**

Timely testing is a key determinant of management outcomes of coronavirus disease 2019 (COVID-19). Real-time reverse transcription polymerase chain reaction tests are currently the mainstay for COVID-19 testing. However, serological point-of-care tests (PoCTs) can be useful in identifying asymptomatic and recovered cases, as well as herd immunity.

**Objective:**

The aim of this study was to assess COVID-19 PoCTs in Kenya to support the emergency use authorisation of these tests.

**Methods:**

Between March 2020 and May 2020, 18 firms, of which 13 were from China, submitted their PoCTs to the national regulatory authority, the Pharmacy and Poison Board, who in turn forwarded them to the Kenya Medical Research Institute for pre-evaluation assessment. The tests were run with real-time reverse transcription polymerase chain reaction COVID-19-positive samples. Pre-COVID-19 plasma samples that were collected in June 2019 were used as negative samples. The shelf lives of the PoCTs ranged from 6 to 24 months.

**Results:**

Only nine (50%) tests had sensitivities ≥ 40% (range: 40% – 60%) and the ability of these tests to detect IgM ranged from 0% to 50%. Many (7/18; 38.9%) of the kits had very weak IgM and IgG band intensities (range: 2–3).

**Conclusion:**

Serological-based PoCTs available in Kenya can only detect COVID-19 in up to 60% of the infected population.

## Introduction

The world is grappling with one of the worst disease pandemics ever experienced in over 100 years, the coronavirus disease 2019 (COVID-19). According to a World Health Organization situation report, as of 07 June 2020, the number of COVID-19 cases had reached 6 931 000 people globally, with more than 400 121 deaths. In Kenya, the number of reported cases as of 06 June 2020 was 2600 with 83 deaths.^1^ Real-time reverse transcription polymerase chain reaction (rRT-PCR) is currently the gold standard for severe acute respiratory syndrome coronavirus 2 (SARS-CoV-2) diagnosis as it is highly sensitive and relatively easy to develop.^2^ However, rRT-PCR test protocols are complex, expensive, mainly suited to advanced laboratories, and typically take 4–6 h to complete. Moreover, these tests are manufactured by predominantly European and American companies that have adopted a ‘me first’ policy due to the high numbers of COVID-19-related deaths in their own countries, thereby successfully eliminating the chances of these test kits flowing to Africa and Kenya in quantities sufficient for use.^3,4^ On the other hand, serological-based point-of-care tests (PoCTs) that take 5–15 min to complete can pick up asymptomatic or recovered cases of COVID-19, making them suitable to support disease surveillance and the determination of herd immunity.^5^ However, these PoCTs have low overall sensitivities (34% – 80%) and specificities (70% – 100%) compared to COVID-19 rRT-PCR as the gold standard.^6^

Serological-based PoCTs detect plasma levels of immunoglobin G (IgG), immunoglobin M (IgM), and, sometimes, IgA against SARS-CoV-2. The levels of IgM are elevated during the first week after SARS-CoV-2 infection, peak at 2 weeks, and then reduce to baseline levels in most patients. On the other hand, IgG is detectable after 1 week and is sustained at a high level for a long period.^7,8,9^ Currently, the World Health Organization does not recommend the use of COVID-19 serological tests in the clinical setting. However, the potential of the use of antigen-based PoCTs at triage to rapidly detect cases is acknowledged.^10^

To evaluate in vitro serological-based PoCTs, the World Health Organization recommends the use of a minimum of 200 prospective COVID-19 specimens (100 confirmed rRT-PCR positive samples and 100 prospective specimens from patients with signs and symptoms suggestive of COVID-19). At least 30 specimens should be rRT-PCR positive at the time of specimen collection and within a week of symptom onset. The remaining data may be supplemented during the review process. For diagnostic specificity, 200 individual specimens from symptomatic patients that tested negative to COVID-19 by rRT-PCR and at least 1000 specimens from the general population collected before November 2019 are used. At least 50% of data are requested for submission.^11^

Manufacturers of commercial COVID-19 PoCTs submit their PoCTs to the Pharmacy and Poisons Board (PPB), Nairobi, Kenya, for evaluation to facilitate and enhance the issuance of emergency use authorisation (EUA). Upon receiving the EUA, the supplier of a PoCT may opt to submit it for the full evaluation.

In the face of the pandemic, the regulatory pathway of EUA bypasses the often longer, data scrutinising pre-evaluation process conducted by the regulator (the PPB) before allowing market authorisation to a manufacturer. Owing to the novelty of the coronavirus and the devastating risk of death of infected persons, the need to avail the PoCTs and rRT-PCR to the general population was paramount. The need for laboratory performance pre-evaluation was identified as a key component required in ascertaining the quality of the PoCTs, thus helping to ensure that in the interim period of use of the PoCTs, performance, efficacy and safety standards are met.

This study aimed to conduct a preliminary evaluation of commercial PoCTs that had been presented to the Kenya Medical Research Institute (KEMRI), Nairobi, Kenya, by the PPB.

## Methods

### Ethical considerations

This study was initially approved by the Scientific Ethical Review Unit at KEMRI under protocol KEMRI/SERU/CBRD/209/4008. It was also reviewed and approved by the Kenyatta National Hospital Ethical Review Committee under protocol number P274/05/2020. COVID-19 patients or subjects had to provide written informed consent before blood sample collection. All samples were anonymised.

### Sample collection

A total of 50 anonymised blood samples were collected through purposive sampling from patients (male and female) of all ages with active infection as determined by COVID-19 rRT-PCR. The study subjects were from the isolation and quarantine centre at the Kenyatta National Hospital Infectious Diseases Unit. After consenting, 5 mL blood samples were drawn from the subjects and transported to the KEMRI Innovation and Technology Division. The serum was separated and stored at –20 °C until the day of the pre-evaluation assessment. Eighteen COVID-19 PoCTs were received from the PPB, the Kenyan drug regulatory authority, who had received the tests from various clients for pre-evaluation assessment and subsequent registration for EUA. The kits included in the study were rapid-format antibody-based PoCTs that were meant to detect IgM or IgM/IgG antibodies to SARS-COV-2 nucleocapsid or spike 1 or 2 proteins. Pre-COVID human de-identified archived blood samples were collected from national blood transfusion centres and were randomly selected for the pre-evaluation of kits. They were stored at –80 °C at the KEMRI Innovation and Technology Division to serve as COVID-negative samples.

### Test evaluation

The tests were run according to the manufacturers’ instructions under a biosafety cabinet. Briefly, for lateral flow assays, the procedure was as follows: blood samples were spun in a centrifuge and the serum and plasma were separated. The test cassette was removed from foil and allowed to equilibrate to room temperature. One drop (about 10 μL) of plasma sample was loaded using a micropipette into the sample well in the cassette. Thereafter, about 60 μL (2–3 drops) of the assay solution (chase buffer) was pipetted into the sample well in the device. After about 10 min, the results were interpreted. Only tests in which the colour of the control line changed were considered valid, and if a coloured line was observed for IgM or IgG, the test was considered positive. The intensity of the colour was compared with that of the colour reference card and semi-quantified. The immunofluorescent protocol was as follows: 150 μL of detector diluent was transferred into a vial containing detector crystal. 10 μL of plasma sample was then added, mixed, and, using a micropipette, 75 μL of the mixture was pipetted into the sample well in the cartridge. After 10 min, the cartridge was placed into the immunofluorescent reader and the results were read and interpreted.

### Determination of sensitivity and specificity of the assays

Data generated were recorded and analysed using Microsoft Excel 2010 (Microsoft Corporation, Redmond, Washington, United States) and STATA version 14.1 (StataCorp LLC, Brownsville, Texas, Unites States). The specificity of the assays was determined using a panel of 50 pre-COVID-19 pandemic serum samples from June 2019. Sensitivity was determined for each assay using 50 rRT-PCR SARS-CoV-2-positive samples. The RNA for confirmatory tests were extracted using a QIAamp Viral RNA mini kit (QIAGEN, Germantown, Maryland, United States). The PCR was done on an Applied Biosystems QuantStudio™ 5 (Thermofisher Scientific, Waltham, Massachusetts, United States) PCR machine using a Sacace Biotechnologies SARS-CoV-2 Real-TM detection kit (Scalabrini, Como, Italy) with their recommended cycling conditions set at 35 °C for 20 min for reverse transcription, initial PCR activation at 94 °C for 10 s, and five cycles at 64 °C for 25 s. The cycling step was done at 94 °C for 10 s followed by 64 °C for 25 s for 45 cycles. Analysis of the results was done using QuantStudio Design and Analysis software (Thermofisher Scientific, Waltham, Massachusetts, United States) and interpreted using the Sacace SARS-CoV-2 Real-TM result analysis guide.

The antibody tests were considered positive when the control band and IgM, IgG or both bands were visible. Diagnostic sensitivity (%) was calculated as *a*/(*a* + *c*) × 100 while diagnostic specificity (%) was calculated as *d*/(*b* + *d*) × 100, where *a* represents true positive results, *c* represents false negatives, *b* represents false positives and *d* represents true negatives.

The PoCTs were considered to have a high sensitivity when able to correctly identify all positive samples in a panel. Tests that only detected 40% or lower of the positive samples in the panel were deemed to have lower sensitivity as they would miss positives and give higher false negative rates.

## Results

The study involved a total of 18 PoCTs from different manufacturers. The number of kits submitted by each manufacturer differed as the national policy on the number of kits required for EUA pre-evaluation assessment had not been established at the time. The shelf lives allocated to the kits were highly varied: 6 months (2/18; 11.1%), 12 months (8/18; 44.4%), 18 months (1/18; 5.6%), and 24 months (7/18; 38.9%) (Table 1). Many (7; 38.9%) of the kits were manufactured in March 2020 (and the study commenced in May 2020). Thirteen (72.2%) of the kits analysed were manufactured in China, 2 (11.1%) in Korea, 1 (5.6%) in Canada, 1 (5.6%) in the United States, and 1 (5.6%) in Malaysia.

**TABLE 1:** Characteristics of COVID-19 point-of-care test kits submitted to the Kenya Medical Research Institute by the Pharmacy and Poisons Board, Nairobi, Kenya, for pre-evaluation assessment, March–May 2020.

Kit identity number	Country of origin	Manufacture date	Expiration date	Shelf life (months)	No. provided
1	Malaysia	Not provided	05 Mar. 2021	12	11
2	China	20 Mar. 2020	19 Mar. 2021	12	100
3	China	10 Mar. 2020	10 Mar. 2021	12	100
4	Korea	Not provided	26 Mar. 2021	12	15
5	Canada	Not provided	Mar. 2022	12	19
6	China	25 Mar. 2020	Mar. 2021	12	11
7	Korea	Not provided	08 Dec. 2021	18	275
8	China	Mar. 2020	Feb. 2022	24	20
9	China	24 Feb. 2020	24 Mar. 2022	24	40
10	China	17 Mar. 2020	17 Mar. 2021	12	25
11	China	06 Mar. 2020	05 Sept. 2020	6	20
12	China	26 Mar. 2020	26 Mar. 2022	24	50
13	China	Not provided	28 Oct. 2020	6	37
14	China	03 Feb. 2020	08 Feb. 2021	12	50
15	China	11 May 2020	29 May 2022	24	500
16	United States	11 May 2020	11 Apr. 2022	24	50
17	China	Not provided	Mar. 2022	24	50
18	China	Not provided	Mar. 2022	24	50

Most of the tests (15/18; 83%) were based on COVID-19 IgG or IgM detection on separate bands; one was based on COVID-19 IgG or IgM detection on a combined band, while two could only detect IgM. The signal from 17 tests could be detected by the naked eye, while the signal of one kit (ID 11) could be detected only using a fluorometric machine which is a semi-automated in vitro diagnostic device that detects analytes through fluorescent scanning.

### Diagnostic sensitivity of the point-of-care test kits

**FIGURE 1:**
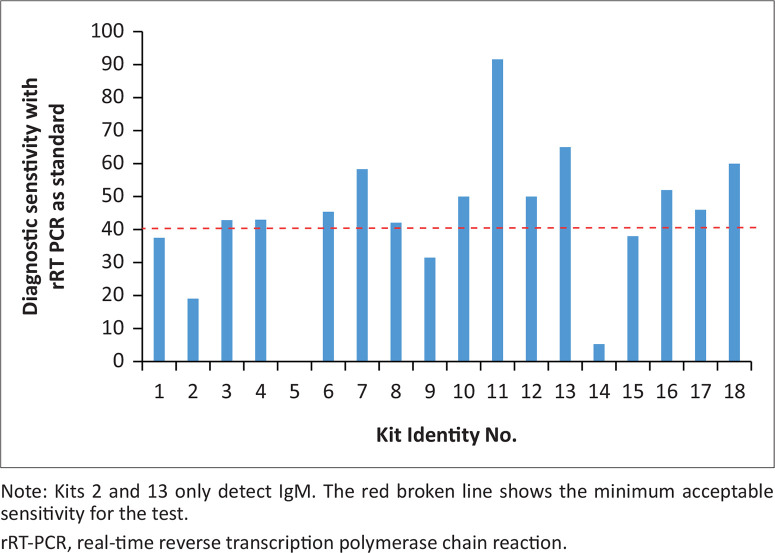
Diagnostic sensitivity of the pre-assessed COVID-19 point-of-care tests submitted to the Kenya Medical Research Institute by the Pharmacy and Poisons Board, Nairobi, Kenya, for pre-evaluation assessment, March–May 2020. Combined IgG and IgM sensitivity was assessed using rRT-PCR as standard.

Kit 2 (IgM) and kit 14 showed low sensitivities of 26.7% and 11.1%; Kit 5 showed no activity; Kit 11, which was the only kit with IgG and IgM combined in a single band test, showed high sensitivity (Figure 1). Of the total 18 kits, 6 (33.3%) kits had sensitivities of at least 50%: kits 7, 10, 12, 13, 16, and 18. Five kits had diagnostic sensitivities between 40% and 50% (kits 3, 4, 6, 8 and 17), while kits 1, 9 and 15 had sensitivities between 30% and 40%. Only kits manufactured in China and the United States managed to score more than 40% diagnostic sensitivity.

### Diagnostic sensitivity of the IgG and IgM separate bands

**FIGURE 2:**
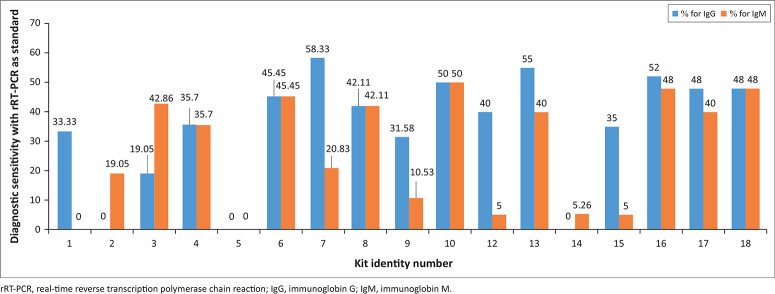
Diagnostic sensitivity of the pre-assessed COVID-19 point-of-care tests submitted to the Kenya Medical Research Institute by the Pharmacy and Poisons Board, Nairobi, Kenya, for pre-evaluation assessment, March 2020 – May 2020. Separate IgG or IgM sensitivities were assessed using rRT-PCR as standard.

The diagnostic sensitivity of the IgG and IgM bands were considered separately for all the PoCTs (Figure 2). The IgM band of kit 1 did not demonstrate any capacity to detect IgM. Kit 2, an IgM-only kit, had a low sensitivity of 19% and a specificity of 95%. The sensitivity of Kit 13 was 55% for IgG and 40% for IgM. Kit 12 showed higher diagnostic sensitivity for IgG (40%) than for IgM (5%). Kit 3 was the only kit that showed higher diagnostic sensitivity for IgM (43%) than IgG (19%). Interestingly, five kits (kits 4, 6, 8, 10, and 18) showed equal diagnostic sensitivity for IgG and IgM. There was a weak positive correlation between the IgM and IgG diagnostic sensitivities of the kits (Pearson’s correlation coefficient [*r*] = 0.2527; *R*^2^ = 0.0639; *p* = 0.45).

### Diagnostic specificity of the point-of-care test kits

Eight kits (kits 1, 3, 8, 9, 12, 14, 16, and 18) had a diagnostic specificity of 100%, while five kits had low diagnostic specificities of 81% (kit 7), 92% (kit 17), 94% (kit 13), and 95% (kits 2 and 15). The remaining five kits were not tested for diagnostic specificity as they had run out of stock.

### IgM and IgG band signal intensities of the point-of-care test kits

**FIGURE 3:**
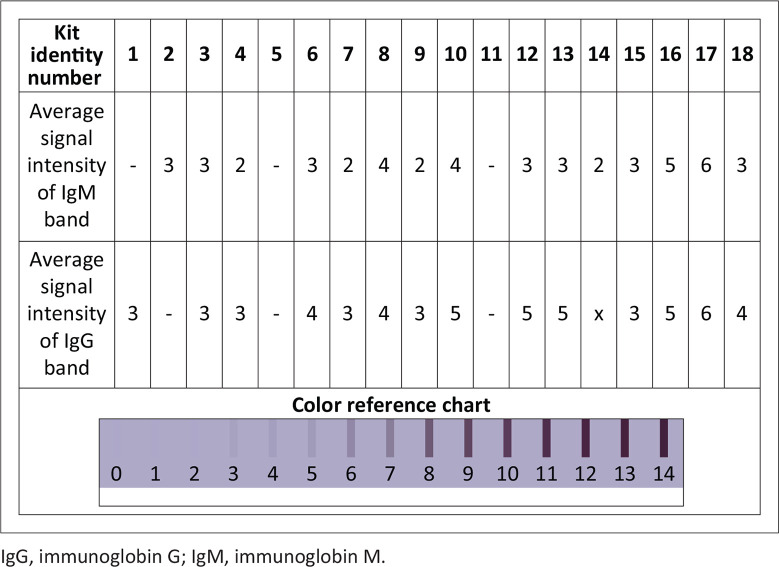
Colour intensity of the positive control signal for the IgM and IgG bands of point-of-care tests assessed at Kenya Medical Research Institute between March 2020 and May 2020.

The intensities of the IgM and IgG bands of the PoCTs were analysed with the assistance of a colour reference chart (Figure 3). The average intensities of the observed bands of many (7/18; 38.8%) of the kits were at score 3 on the scale (Figure 3). The intensities of the IgG bands were generally stronger than those of IgM. Kits 3, 6, 8, 10, 12, 13, 16, 17, and 18 displayed the strongest intensities, which corresponded with their higher diagnostic sensitivities compared to the remaining kits.

## Discussion

This study carried out a pre-evaluation assessment of PoCT kits submitted by manufacturers to the PPB for pre-evaluation. Out of the kits evaluated, 50% had sensitivities of ≥ 40%, and most of the kits had low band intensities. The kits with sensitivities of ≥ 40% were given emergency approval. Currently, rRT-PCR tests are the mainstay of SARS-CoV-2 diagnosis; however, they have complex protocols, are expensive, and require advanced laboratory equipment.^2^ The main goal of introducing a PoCT is to avail quick test results to the healthcare workers or the patient to support fast clinical management decisions and, ultimately, improve patient outcomes and overall public health. It is hoped that PoCTs will be useful in identifying asymptomatic and recovered cases of COVID-19 as well as herd immunity.^12^

The pre-evaluation assessment of PoCTs is crucial to mitigating the risks associated with the introduction of kits that can cause public anxiety and false alarm to the market. The regulatory pathway for the evaluation and assessment of PoCTs per the stipulated turnaround time for in vitro diagnostics takes 1–3 years; however, the COVID-19 pandemic presented increased pressure and the need to grant marketing authorisation to manufacturers, necessitating the development of a pre-evaluation assessment policy. According to unpublished reports from the PPB, and as a pre-requisite measure for issuance of the EUA, those kits without satisfactory sensitivity and specificity results must be subjected to further evaluation.

The shelf lives of the PoCTs in this study ranged from 6 to 24 months. The manufacturers did not provide reports of their stability data or their plans for generating such data.^13^ The study showed that 50% of the PoCTs that were submitted for pre-evaluation assessment did not meet the criteria to proceed to full evaluation as they were unsatisfactory. Of the 18 PoCTs, one had no activity at all, two IgM-detecting tests had both low sensitivity and low specificity, and three tests displayed high false-positive values. Only nine (50%) tests had sensitivities of ≥ 40% (range: 40% – 60%). The capacity of these nine tests to detect IgM ranged from 0% to 50%. The majority (57.1%) of the tests displayed very weak visual IgM band intensities (scale scores of 2–3).

The sensitivities observed in this evaluation were lower than those reported elsewhere, and the ability of the tests to detect IgM varied, with one test failing to capture any COVID-19 IgM. There was also a weak positive correlation between IgM and IgG sensitivity for the kits. The Abbott ID NOW COVID-19 test (Abbott Laboratories, Abbott Park, Illinois, United States) is said to be the most sensitive and specific PoCT on the global market, with a sensitivity of 80.4% and specificity of 95.9% with rRT-PCR as the standard.^14,15^ A study conducted to assess the quality of a PoCT based solely on PCR-positivity among anonymous blood donors in Uppsala University, Uppsala, Sweden, reported a high performance, with sensitivities of 69% for IgM and 93.1% for IgG, and specificities of 100% for IgM and 99.2% for IgG.^16^ Another study conducted in China among COVID-19-positive patients established the sensitivity and specificity of the test they assessed to be 88.66% and 90.63%.^17^ A study done at Guangzhou Eighth People’s Hospital, China, among patients with confirmed SARS-CoV-2 infection found that the combined detection of nucleocapsid-specific and spike-specific IgM and IgG could identify up to 75% of SARS-CoV-2-positive cases in the first week using an enzyme-linked immunosorbent assay test kit.^18^ In consonance with the findings of this study, the Organization for Economic Co-operation and Development has emphasised the need to recognise that PoCTs have not been fully developed for SARS‑CoV‑2 and their true clinical performance is mostly unknown.^19^

A satisfactory quality assessment of COVID-19 diagnostics is very critical. Recently, quality failure-related issues have been reported globally. These include instances such as India stopping the use of 500 000 PoCTs from two Chinese firms for questionable efficacy,^20^ a report of initial PCR test kits that were contaminated with coronavirus synthetic materials in the United States,^21^ Britain buying millions of PoCTs that did not work,^22^ first samples of PoCTs manufactured in India failing the quality tests,^23^ and Spain withdrawing Chinese-manufactured kits with a sensitivity of 30%.^24^ It is noteworthy that kit 11, which failed in this study, is from the same company that made the kits that failed in India.

All kits need to be re-evaluated using standard COVID-19 IgG and IgM to establish a gold standard PoCT. The kits assessed in this pre-evaluation should be limited to use in research and sero-surveillance only. Furthermore, there is a need for continued evaluation of these kits using whole blood samples with larger sample sets of at least 400 COVID-19-positive and negative samples. This would be a full evaluation of the kits and would increase the power of the study. There is also a need to formulate national and international policies on the determination of shelf lives of products that are developed during medical emergencies such as the COVID-19 pandemic.

### Limitations

This study only used plasma matrix. There is a need to assess the performance of these PoCT kits using whole blood samples, especially whole blood from finger pricks and serum as per the manufacturers’ instructions. The sample size used in this study was less than the 400 samples recommended by the World Health Organization. The suppliers of the PoCTs could not provide the adequate number of kits required.

### Conclusion

There was very poor IgM detection by most of the PoCT kits assessed and this could affect timely detection of COVID-19 early infection and spread as compared to rRT-PCR. Similarly, the serological-based test kits available in the country can only detect up to 60% of the infected population.

## References

[1] John Hopkins University. Epidemic map updated in real-time. Baltimore, MD: Center for Systems Science and Engineering; 2020.

[2] Jeong Sae-im. Korea approves 2 more COVID-19 detection kits for urgent use. Korea Biomed Rev. 2020.

[3] Liang M, Du L, Liu J, et al. SARS patients-derived human recombinant antibodies to S and M proteins efficiently neutralize SARS-coronavirus infectivity. Biomed Environ Sci. 2005;186(6):363. 16544518

[4] Sheridan C.Coronavirus and the race to distribute reliable diagnostics. Nat Biotechnol. 2020;38:382–384. 10.1038/d41587-020-00002-2 32265548

[5] Hopkins J. Serology-based tests for COVID-19. Baltimore, MD: Johns Hopkins; 2020.

[6] Zainol R, Othman S, Abdul S, et al. Diagnostic performance of COVID-19 serology assays. Malaysian J Pathol. 2020;42(1):13–21. 32342927

[7] Vabret N, Graham J, Conor G, et al. Immunology of COVID-19 current state of the science. Immunity. 2020;42(2):910–941. 10.1016/j.immuni.2020.05.002 PMC7200337 32505227

[8] Srikrishna D, Dhillon RS, Beier D. We need a cheap way to diagnose coronavirus. Harv Bus Rev. 2020.

[9] Hou H, Wang T, Zhang B, et al. Detection of IgM and IgG antibodies in patients with coronavirus disease 2019. Clin Translational Immunol. 2020;9(5):e1136. 10.1002/cti2.1136 PMC7202656 32382418

[10] World Health Organization. Advice on the use of point-of-care immunodiagnostic tests for COVID-19: Scientific brief. Geneva: World Health Organization; 2020.

[11] World Health Organization. Instructions for submission requirements: In vitro diagnostics (IVDs) detecting antibodies to SARS-CoV-2 virus. Geneva: World Health Organization; 2020.

[12] Drain P, Hyle E, Noubary F, et al. Diagnostic point-of-care tests in resource-limited settings. Lancet Infect Dis. 2014;14(3):239–249. 10.1016/S1473-3099(13)70250-0 24332389 PMC4016042

[13] World Health Organization (b). Establishing stability of in vitro diagnostic medical devices. (No. WHO/BS/2017.2304). Geneva: WHO; 2019.

[14] I. N. COVID-19. Abbott [homepage on the Internet]. [cited 2020 June 14]. Available from: https://www.alere.com/en/home/

[15] Hogan C, Sahoo M, Huang C, et al. Five-minute point-of-care testing for SARS-CoV-2 not there yet. J Clin Virol. 2020;128:104410. 10.1016/j.jcv.2020.104410 32403009 PMC7194071

[16] Hoffman T, Nissen K, Krambrich J, et al. Evaluation of a COVID-19 IgM and IgG rapid test; an efficient tool for assessment of past exposure to SARS-CoV-2. Infect Ecol Epidemiol. 2020;10(1):1754538. 10.1080/20008686.2020.1754538 32363011 PMC7178815

[17] Li Z, Yi Y, Luo X, et al. Development and clinical application of a rapid IgM-IgG combined antibody test for SARS-CoV-2 infection diagnosis. J Med Virol. 2020;92(9):1518–1524. 10.1002/jmv.25727 32104917 PMC7228300

[18] Sun B, Feng Y, Zheng P, et al. Kinetics of SARS-CoV-2 specific IgM and IgG responses in COVID-19 patients. Emerg Microb Infect. 2020;9(1):940–948. 10.1080/22221751.2020.1762515 PMC7273175 32357808

[19] OECD. Home testing for COVID-19: A way to lift confinement restrictions. OECD. Paris; 2020.

[20] Hindustan Times New Delhi China. Concerned as India decides to stop use of Chines COVID-19 test kits. New Delhi; 2020.

[21] New York Times. CDC Labs were contaminated delaying corona virus testing official say. New York; 2020.

[22] The Sunday Times. Britain has millions of coronavirus antibody tests, but they don’t work. London; 2020.

[23] Nidheesh LA. First samples of HLL’s India-made rapid testing fail quality test in Kerala. New Delhi: LiveMint; 2020.

[24] The Guardian. Coronavirus test kits withdrawn in Spain over poor accuracy rate. Madrid; 2020.

